# Pathogenicity of Isolates of *Serratia Marcescens* towards Larvae of the Scarab *Phyllophaga Blanchardi* (Coleoptera)

**DOI:** 10.3390/pathogens4020210

**Published:** 2015-05-13

**Authors:** Mónica L. Pineda-Castellanos, Zitlhally Rodríguez-Segura, Francisco J. Villalobos, Luciano Hernández, Laura Lina, M. Eugenia Nuñez-Valdez

**Affiliations:** 1 Centro de Investigación en Dinámica Celular, Instituto de Ciencias Básicas y Aplicadas, Universidad Autónoma del Estado de Morelos, Av. Universidad 1001, Col. Chamilpa, CP 62209, Cuernavaca, Morelos, Mexico; E-Mail: mlpc@uaem.mx; 2 Centro de Investigación en Biotecnología, Universidad Autónoma del Estado de Morelos, Av. Universidad 1001, Col. Chamilpa, 62209, Cuernavaca, Morelos, Mexico; E-Mails: zit.rodriguez@gmail.com (Z.R.-S.); llina@uaem.mx (L.L.); 3 El Colegio de la Frontera Sur, Carretera Panamericana y Periférico Sur s/n Barrio María Auxiliadora, CP 29290, San Cristóbal de Las Casas, Chiapas, Mexico; E-Mail: fvillalobos@ecosur.mx; 4 Facultad de Química, Universidad Nacional Autónoma de Mexico, Mexico D.F., Mexico; E-Mail: lhergom@unam.mx

**Keywords:** *Serratia marcescens*, white grub, pathogenicity, *Phyllophaga*, scarab, biological control, insecticidal protein, serralysin

## Abstract

*Serratia marcescens* is a Gram negative bacterium (Enterobacteriaceae) often associated with infection of insects. In order to find pathogenic bacteria with the potential to control scarab larvae, several bacterial strains were isolated from the hemocoel of diseased *Phyllophaga* spp (Coleoptera:Scarabaeidae) larvae collected from cornfields in Mexico. Five isolates were identified as *Serratia marcescens* by 16S rRNA gene sequencing and biochemical tests. Oral and injection bioassays using healthy *Phyllophaga blanchardi* larvae fed with the *S. marcescens* isolates showed different degrees of antifeeding effect and mortality. No insecticidal activity was observed for *Spodoptera frugiperda* larvae (Lepidoptera: Noctuidae) by oral inoculation. *S. marcescens* (Sm81) cell-free culture supernatant caused significant antifeeding effect and mortality to *P. blanchardi* larvae by oral bioassay and also mortality by injection bioassay. Heat treated culture broths lost the ability to cause disease symptoms, suggesting the involvement of proteins in the toxic activity. A protein of 50.2 kDa was purified from the cell-free broth and showed insecticidal activity by injection bioassay towards *P. blanchardi*. Analysis of the insecticidal protein by tandem- mass spectrometry (LC-MS/MS) showed similarity to a Serralysin-like protein from *S. marcescens* spp. This insecticidal protein could have applications in agricultural biotechnology.

## 1. Introduction

Sustainable agriculture is one of the goals of modern society. The lack of sufficient product input for agriculture combined with a low impact on the environment is a limiting factor to increase production without harming the environment. The use of entomopathogenic bacteria as bioinsecticides has been shown as a promising alternative to the use of chemicals for the control of insect pests and therefore to increase production. Besides, the use of bacterial insecticidal toxins such as the delta-endotoxin from *Bacillus thuringiensis* has been proven to be a successful strategy to cope with insect pests [1]. However, there are still many insect pests that are not affected by the available *B. thuringiensis* toxins. In addition the development of resistance to *B. thuringiensis* toxins is a special constraint. Additionally, the search and characterization of new pathogenic bacteria and insecticidal toxins are important in order to cope with this constraint. The possibility to use several insecticidal toxins with a different mode of action in the same insect pest, in parallel to *B. thuringiensis* toxins, is suggested as a strategy to decrease the speed of resistance development [2]. This strategy could also increase or synergize insecticidal activity. The discovery of new toxins could be very useful for the control of insects not susceptible to the available *B. thuringiensis* toxins.

In Mexico, there are 68 species of “white grubs” reported as potential pests [3], and there is no efficient biological control agent to cope with them. White grubs are the larval stages of scarabs living in the soil for long periods of time feeding off of the roots of plants. They cause severe damage to many important crops worldwide. Therefore, it is relevant to isolate bacterial strains with insecticidal activity for the control of these larvae. Recently, a Mexican *S. entomophila* strain active against larvae of several species of *Phyllophaga* was reported [4]. The bacteria cause an antifeeding effect and mortality by oral and injection bioassays either by the bacteria themselves or by cell-free culture supernatants. It has been proposed that the production of toxins by *S. entomophila* and other *Serratia* spp act at different moments of the infection process, either by acting on the epithelial midgut cells at the beginning of the infection or by acting at the hemocoel later in the process, with toxic activities that lead to the death of the insect. A previous report demonstrated [5] the isolation of several pathogenic bacteria from the hemocoel of *Phyllophaga* spp larvae showing evident disease symptoms. In this paper we show the species identification and characterization of five *S. marcescens* strains, some of which have strong toxic activity towards *P. blanchardi*, either by oral or injection bioassay. *S. marcescens* is a Gram negative bacterium classified in the large family of Enterobacteriacea. It is a soil inhabitant that has often been associated with infection of insects [6,7]. A Serralysin-like protein of 50 kDa, isolated from the cell-free culture broth from a *S. marcescens* isolate, was found to be insecticidal to *Phyllophaga* spp. The 50 kDa Serralysin-like protein might be a good candidate to address for biotechnological applications focusing on integrated pest management of *Phyllophaga* spp. This is the first report on the identification of a Serralysin-like protein with insecticidal activity towards *P. blanchardi* larvae.

## 2. Results and Discussion

### 2.1. Identification of Pathogenic S. marcescens Strains

A search for entomopathogenic bacteria was conducted taking samples from the hemocoel of dead third-instar larvae presenting previous disease symptoms as darker color, softer appearance and decrements in the amount of food consumed compared to healthy larvae. Ninety-six bacterial isolates, coming from a total of 116 larvae, had been obtained previously [5]. Twelve bacterial isolates were selected based on the fact that the original hemocoel samples contained high bacterial titers (1 × 10^9^ bacteria mL^−1^) and each sample presented bacteria with a similar colony morphology. The other 84 hemocoel samples presented a mixture of bacteria with different colony morphology and showed lower bacterial titers (1 × 10^2^ bacteria mL^−1^). Only five *S. marcescens* strains were found among the 12 isolates. The five strains were isolated from *Phyllophaga ravida* larvae.

**Figure 1:**
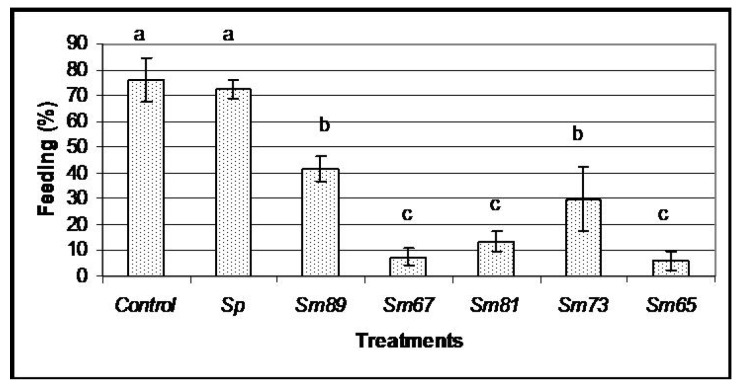
Feeding behavior of *S. marcescens* isolates towards larvae of *P. blanchardi* by oral inoculation. The percentage of feeding represents an average of food ingested during 6 days of oral inoculation. Control groups were fed with uncoated pieces of carrot and also carrot coated with the non-pathogenic bacterium *Serratia plymuthica* ATCC15928. A lack of significant differences is indicated by the same letter above the bars (ANOVA, *p* < 0.001); *n* = 10.

Oral bioassays were done to test the ability of the bacterial isolates to cause pathogenic symptoms: antifeeding effect, change in color and mortality. Healthy larvae of *P. blanchardi* were fed with small pieces of carrot containing the *S. marcescens* isolates, as indicated in the Materials and Methods section. Larvae in the control treatment group were fed with un-inoculated carrot. A second negative control was done with larvae fed with the non-pathogenic bacteria *S. plymuthica* ATCC 15928. As indicated in Figure 1, the bacterial isolates of *S. marcescens* caused different degrees of antifeeding effects, since the larvae showed a significant decrement in the amount of food consumed compared to the control larvae (*p <* 0.05) during the 6 days of oral inoculation. The larvae fed with the isolates of *S. marcescens* 67 (Sm67), *S. marcescens* 81 (Sm81) and *S. marcescens* 65 (Sm65) consumed only a small proportion of the available food, showing a food consumption of 7.51%, 13.46% and 6.06% respectively; the strains *S. marcescens* 89 (Sm89) and *S. marcescens* 73 (Sm73) consumed only 41.84% and 29.89% of their food, respectively. Larvae from the control groups, fed with un-inoculated carrot and with the nonpathogenic bacteria *S. plymuthica* consumed 76.19% and 72.33% of the available food, respectively. After the inoculation period of 6 days, all the larvae were fed with inoculated carrot. The larvae treated with the different isolates of *S. marcescens* maintained low levels of food consumption compared to control larvae, even after the pathogenic bacteria was separated from the food; the larvae in this control group consumed about 75% of the available carrot, and the average of food consumption in the larvae fed with the *S. marcescens* isolates was 25%.

**Figure 2:**
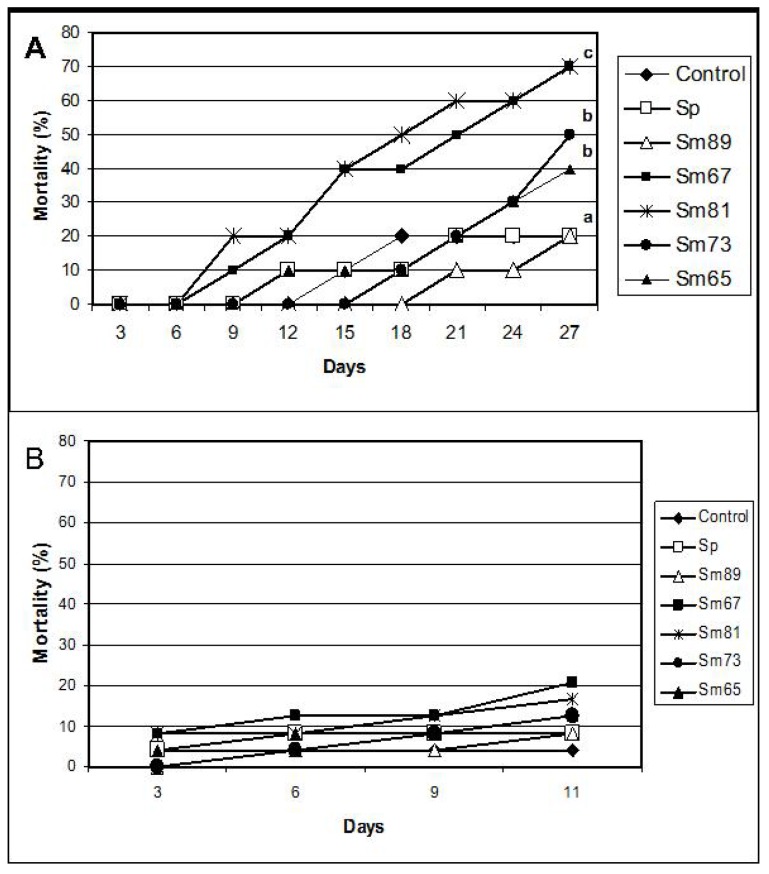
Mortality of larvae fed with the *S. marcescens* isolates. Panel A. *P. blanchardi*. Mortality was recorded during 27 days after oral inoculation. Control groups were fed with pieces of carrot alone and also with carrot containing the non-pathogenic bacterium *Serratia plymuthica* ATCC15928. No significant differences are indicated by the same letter above the lines (χ^2^, *p* < 0.01); *n* = 10. Panel B. *Spodoptera frugiperda.* Mortality was recorded during 11 days. Control groups were fed with an artificial diet where bacteria were replaced with the same amount of sterile water and *S. plymuthica* ATCC15928. No significant differences between treatments were observed (χ^2^, *p* < 0.05).

Significant mortality (*p <* 0.01) compared with control larvae was observed for the larvae inoculated with Sm67, Sm81, Sm73 and Sm65 after 27 days from the beginning of the test (Figure 2). The observed mortality for the control larvae was 20% and the observed mortality in the group fed with the Sm89 isolate was also 20%. On the contrary, the larvae inoculated with the other four *S. marcescens* isolates showed a mortality of 70% for both Sm67 and Sm81 and mortalities of 50% and 40% for Sm73 and Sm65, respectively. Larvae inoculated with the different *S. marcescens* isolates developed a slightly brown color compared with the beige appearance of healthy larvae observed in the control groups.

To evaluate the potential insecticidal activity of the *S. marcescens* isolates for *P. blanchardi* by intracoelomic inoculation, bioassays were done by injecting 10^3^ bacteria per larva. All five *S. marcescens* isolates caused death of 100% of larvae in a time period of 48 h to 96 h from inoculation. The strain Sm81 killed all larvae after 48 h and Sm73, Sm67 and Sm65 after 72 h from inoculation. The strain Sm89 killed 100% of the larvae after 96h. The same amounts of *E. coli* Top10 and *E. coli* Epi300 injected into the larvae did not cause significant mortality (0%–20%; *p <*0.001) after 96 h from inoculation. Attempts to determine the median lethal dose were not successful, since no differences in larvae mortality were observed at bacterial concentrations higher than 10^3^ bacteria per larva, that is, a concentration between 10^3^ to 10^6^ bacteria per larva caused a mortality of 100% after 48–96 h depending on the strain. Bacterial concentrations lower than 10^3^ bacteria per larva produced no significant mortality, even after 20 days from inoculation. It is possible that once the insect immune response is surpassed by the bacterial infection, larval death occurs very fast.

To test the ability of the *S. marcescens* isolates to kill larvae belonging to a different insect orders, oral bioassays were performed with larvae of *Spodoptera frugiperda* (Lepidoptera: Noctuidae) fed with the five *S. marcescens* isolates. No significant mortality was observed in this bioassay (Figure 2).

### 2.2. Taxonomic Determination of Pathogenic Strains

To define the taxonomic identification of the five isolates (89, 67, 81, 73 and65), the 16S rRNA gene was amplified and sequenced as described in the Materials and Methods section. The sequences were compared with other bacterial sequences using the basic local alignment search tool (BLAST) sequence homology searches at the National Center for Biotechnology Information [8]. The five sequences produced significant alignments (99%) to *S. marcescens* 16S rRNA gene of type strain *S. marcescens* WW4 sequence ID: ref|NR_102509.1| (Figure S1. Supplementary material). Phenotypic and biochemical tests done to characterize the bacterial isolates showed that the five isolates were very similar to *S. marcescens* ATCC13082 (Table S1. Supplementary material).

### 2.3. Identification of Toxic Activity in Sm81 Culture Supernatant

It has been suggested that some extracellular toxin proteins might be involved in *Serratia* spp pathogenicity [4]. To test the ability of the Sm81 extracellular proteins to produce mortality, oral bioassays were done with pieces of carrot containing cell-free supernatant from the Sm81 isolate grown for 24 h using 2 µg of protein per larva. To investigate the nature of the potential toxin-like compounds, a sample of the Sm81 culture broth was also evaluated after being boiled for 5 min. Since proteins are denatured by heat, a decrement in the toxic activity would suggest that a protein would be involved in the toxic activity. Carrot containing nutrient broth with 2 µg of bovine-serum albumin (BSA) was used to feed control larvae. The samples were fed to *P. blanchardi* larvae as indicated in Materials and Methods.

**Figure 3:**
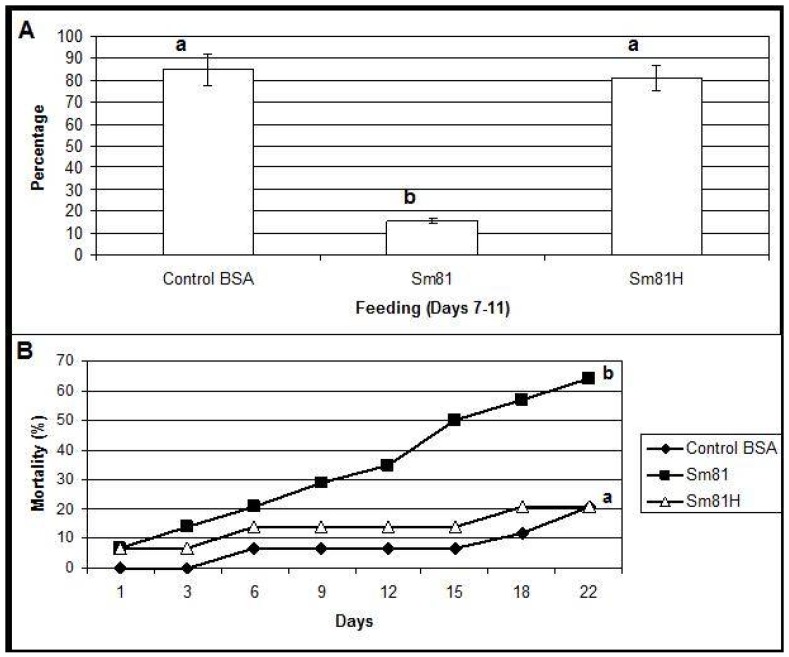
Oral bioassay showing the antifeeding effect (Panel A) and mortality (Panel B) of cell-free culture supernatants from Sm81 isolate towards *P. blanchardi* larvae. The control group was fed with carrot containing nutrient broth plus BSA. The feeding activity of larvae was evaluated during Days 7–11 after an inoculation period of 6 days. The average of food ingestion during the period is shown. Mortality was evaluated during 22 days from the beginning of bioassay. Differences among the groups of larvae shown by the letters above the bars or lines were evaluated by ANOVA for feeding activity and χ^2^ for mortality. Sm81H represents a treatment where the cell-free culture supernatant from the isolate Sm81 were boiled for 5 min. The groups of larvae evaluated are shown by different letters above the lines; *n* = 14.

During the first time period analyzed (inoculation period), from the beginning of the test to Day 6, control larvae and those fed with the boiled sample consumed about 94% and 90% of their food, respectively. A slight but significant decrement (18%, compared to the control 94%) in food consumption was observed in the larvae fed with the Sm81 broth (76%). During the second period analyzed, from Days 7–11, the control larvae showed a food consumption of 85 % (Figure 3). On the other hand, the larvae fed with Sm81 broth consumed only about 15% (*p <* 0.001) of the available food; thereby showing an antifeeding effect. This effect was not observed when the sample was boiled, since larvae showed a food consumption of 81%, which was not significant when compared to the control larvae (85%, *p >* 0.05), but it was significant when compared with the larvae fed with non-boiled broth (15%, *p <* 0.01). The Sm81 broth sample also caused a significant mortality rate of 64% (*p >* 0.05) after 22 days from the beginning of the test. No significant mortality was observed for control larvae and those fed with boiled broth (20%).

**Figure 4:**
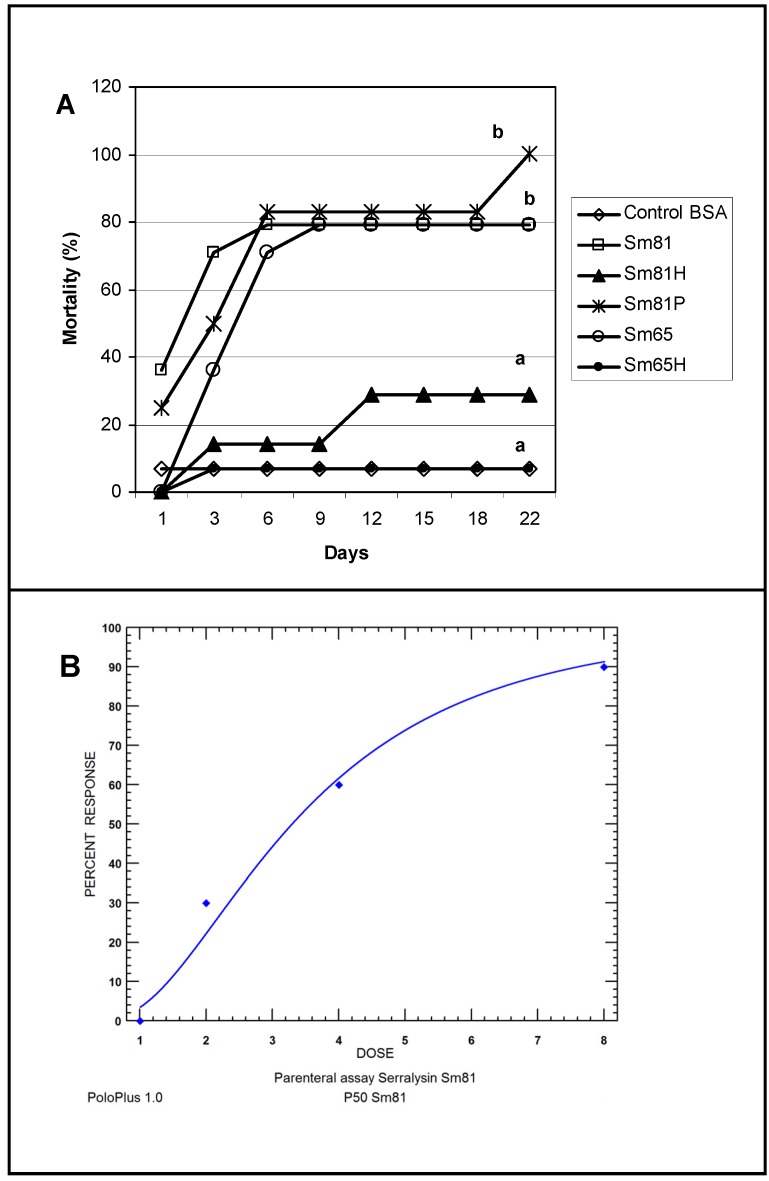
Injection bioassay showing insecticidal activity in the Sm81 and Sm65 cell-free culture supernatant and Sm81 50 kDa protein. Panel A. Supernatant from 24 h culture from the *S. marcescens* 81 and *S. marcescens* 65 (2 µg·mL^−1^) was injected into the larvae. Sm81H and Sm65H represent the same culture supernatant from Sm81 and Sm65 but boiled for 5 min. Sm81P represents the mortality observed for the larvae injected with the 50 kDa protein (2 µg·mL^−1^) purified from the cell-free culture supernatant from Sm81. The control represents a nutrient broth containing BSA (2 µg mL^−1^). Mortality was recorded for 22 days after injection. Differences among the groups of larvae shown by the letters above the lines were evaluated using χ^2^ (*p <* 0.0001; *n* = 14). Panel B. Dose-response bioassay showing dose dependency of the Sm81P50 insect killing activity. Dose-response curve for the injection bioassay of Sm81P50 (1 µg to 8 µg) against *P. blanchardi* larvae. Ten larvae were used for each dose.

To test the ability of the *S. marcescens* extracellular proteins to produce mortality by intracoelomic inoculation, a bioassay by injection was performed. Cell-free culture supernatant from the isolates Sm81 and Sm65 grown for 24 h was injected into healthy *P. blanchardi* larvae. As observed in Figure 4, control larvae injected with nutrient broth containing BSA cause no significant mortality. On the other hand, larvae injected with Sm81 and Sm65 cell-free culture supernatant showed a significant mortality of about 78% (*p <* 0.0001). No significant mortality was observed when the culture broth from both isolates was boiled and injected into the larvae.

### 2.4. A 50 kDa Serralysin-Like Protein Is Responsible for Insecticidal Activity in Sm81

**Figure 5:**
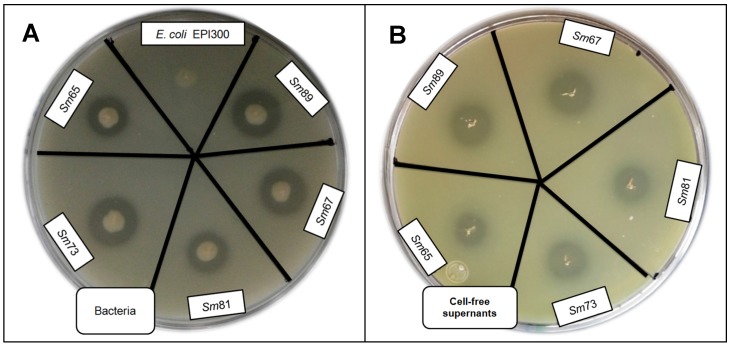
Skim milk plate assay to evaluate protease activity by *S. marcescens* strains and cell-free culture supernatants. Panel A. Protease activity of bacteria. Panel B. Protease activity of cell-free culture supernatants.

It has been reported that a 61 kDa protease from *S. marcescens* HR3 culture broth has insecticidal activity towards the grassland locust *Myrneleotettix palpalis* Zub [9]. A characteristic of the genus *Serratia* is the production of extracellular proteases [6], hence, it was hypothesized that the Sm81 protease was the causative agent for the insecticidal activity observed for the cell-free culture supernatants. The *S. marcescens* isolates and cell-free supernatants were screened for extracellular protease activity on agar-milk plates as reported [10]. Milk clearing zones were produced by the five isolates as expected (Figure 5) demonstrating proteolytic activity. It was observed that the halo diameter sizes of one colony grown for 24 h were about 10–12 mm (Table 1).

**Table 1:** Protease activity of *S. marcescens* bacteria and supernatants.

	Bacteria Halo (mm)			Supernatant Halo (mm)		
Strain	Mean	SD	Confidence limits	mean	SD	Confidence limits
Sm89	12 a	±0.7071	(11.22–12.88)	10.8 a	±1.095	(9.44–12.16)
Sm67	12 a	±0.7071	(11.22–12.88)	11.2 a	±0.4472	(10.64–11.75)
Sm65	10 b	±0.70.71	(9.12–10.88)	8.8 b	±0.4472	(8.24–9.35)
Sm81	10.8 b	±0.4472	(10.24–10.87)	8.6 b	±0.5477	(7.92–9.28)
Sm73	12.2 a	±0.4472	(11.64–12.75)	9.8 a	±0.8367	(8.76–10.84)

Single colonies were grown on nutrient broth agar supplemented with milk. Halo diameters after 24 h are reported in millimeters. Similar letters denots no statistical difference. Differences in halo size were evaluated by ANOVA. Data are representative of five samples per strain.

The halo diameter observed for Sm65 and Sm81 were smaller (10 and 10.8 mm) than the halo diameter observed for Sm89, Sm67 and Sm73 (12 mm). Similar results were observed for the halo diameter of the cell-free supernatants. Five µL of cell-free supernatant produced a halo diameter of about 8–11 mm, and the halos observed for Sm65 and Sm81 were smaller (8.8 and 8.6 mm) than the ones of Sm89, Sm67 and Sm73.

**Figure 6:**
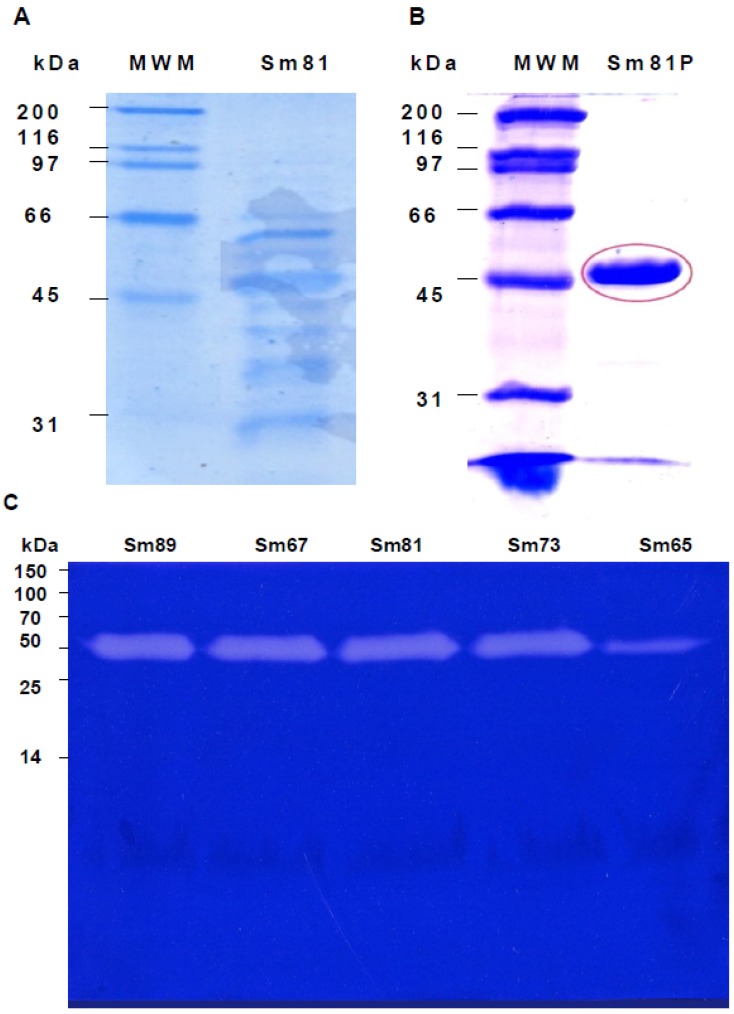
Electrophoretic analysis of *S. marcescens* 81 cell-free culture supernatants. Panel A. Sodium dodecyl sulfate polyacrylamide gel electrophoresis (SDS-PAGE) (12%) analysis of cell-free culture supernatant of *S. marcescens* 81 strain. Cell-free culture supernatant was concentrated and adjusted to 30 µL per line. Panel B. SDS-PAGE analysis of the Sm81 50 kDa protein purified by HiTrap Q FF ion exchange column. Twenty µg of Sm81P50 was charged in the gel line. Panel C. Zymography showing the 50 kDa protease from the *S. marcescens* strains cell-free culture supernatants. Gelatin-SDS-PAGE stained with coomassie brilliant blue. The protein concentration was adjusted to 6 µg per line. For Sm65 the protein concentration was 0.6 µg per line.

In a next step, in order to study whether the *S. marcescens* isolates produced one or several protease(s) of similar or different molecular size, the *S. marcescens* cell-free culture supernatants were analyzed by *in gel* zymography as reported [11]. It was observed in the zymography (Figure 6) that the five *S. marcescens* isolates produced a single band with protease activity, corresponding to an extracellular protease of about 50 kDa (Figure 6, Panel C). No other protein with protease activity was detected by this system.

In order to determine whether the protease isolated from the Sm81 strain presented toxic activity towards *P. blanchardi*, the 50 kDa protein showing protease activity was purified from the Sm81 cell-free culture supernatant (Figure 6, Panel B) as indicated in Materials and Methods and tested for toxic activity by injection bioassays. As indicated in Figure 4 (Panel A), the 50 kDa protein showed strong insecticidal activity, causing a mortality of 83% in *P. blanchardi* larvae. The mortality observed was comparable to the mortality observed for the Sm81 and Sm65 supernatants (79%) from Day 5 to Day 18 after injection. At Day 22 after injection, 100% mortality was observed in the larvae group injected with Sm81P50, contrary to a mortality of 79% observed for Sm81 and Sm65 supernatants at Day 22. It was expected that the purified toxin might cause higher mortality than the mixture of proteins present in the cell-free supernatants. This expectation was only evident at Day 22 after injection.

To determine whether the mortality caused by Sm81P50 was dose-dependant, an injection bioassay administrating several Sm81P50 doses to *P. blanchardi* larvae was done. As observed in Figure 4, Panel B, larvae mortality shows Sm81P50 dose-dependency. The results indicates that mortality increases according to increments in the concentration of purified Sm81P50 administrated to the larvae and also that Sm81P50 is able to kill a high percentage of *P. blanchardi* larvae (90%) after a single injection of 8 µg protein per larva. A similar amount of BSA injected into the larvae used as control for this experiment did not cause mortality, showing that Sm81P50 was responsible for the observed mortality. The LD_50_ value calculated by probit analysis (Figure 4, Panel B) was 3.291 µg (2.292 to 4.806; 95% confidence limit).

**Figure 7:**
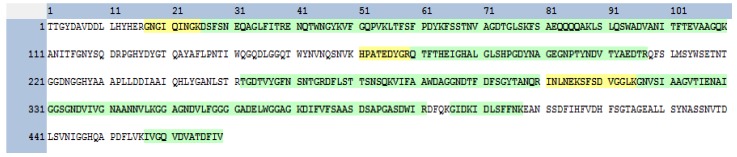
Amino acid sequence of the Serralysin protein (Accession V9NCZ4) showing homology to 22 peptides from Sm81P50. Amino acids shaded in green represent identical peptides that were identified by mass spectrometry. Amino acids shaded in yellow represent peptides also identified by mass spectrometry but not identical to the V9NCZ4 Serralysin.

Analysis of the insecticidal protein by tandem- mass spectrometry (LC-MS/MS) showed a similarity to a Serralysin-like protein from the *Serratia* spp protein group accession A8G880; [12]. Analysis of the tryptic digest of Sm81P50 identified 22 peptides that covered 65.2% of the homolog protein Serralysin (Accession V9NCZ4), with a molecular weight of 50.2 kDa, including peptides from the central region of the protein and the C termini (Figure 7).

A blast homology [8] search of the putative Sm81P50 Serralysin (V9NCZ4) revealed a high degree of homology with other *S. marcescens* serralysin-like proteins. There were about 63 entries with sequences producing significant alignments with identities above 50%. Seven entries were associated with insecticidal proteins, although four of them were partial sequences and were not compared. The insecticidal proteins led to identities of 99%, 99% and 97%, respectively to Serralysin-like proteases from *S. marcescens* strain HR-3 (Sequence ID: gb|ABK55613.1|), *S. marcescens* strain Zhen Jiang (Sequence ID: gb|ACH90152.1|) and *S. marcescens* strain DOAB 216-82 (Sequence ID: gb|AGG14207.1|) corresponding to proteins of 487 amino acids (E-value 0.0) (S2. Supplementary material). Serralysins are Zinc-metalloproteases, which are common in pathogenic bacteria, and are often attributed roles in virulence. These virulence roles include direct toxin activity, processing of other enzymes and degradation of host connective tissues [13]. Complete genomes of the *S. marcescens* strains Db11, WW4, SM39 and FG194 have been announced (National Center for Biotechnology Information [8]), and the 50 kDa protein also leads to significant alignments with Serralysin-like sequences from Db11, WW4, SM39 strains (S.3. Supplementary material). Contrarily, no significant alignment was observed for Serralysin (Accession NC_020064.1) from *S. marcescens* FG194. Alignment of putative Sm81P50 *versus* Db11 Serralysin (Accession WP_038629638.1) led to 56% identity (199 out of 350 amino acids; 2e-127), Sm81P50 *versus* WW4 Serralysin (Accession WP_015377863) led to 63% identity (231 out of 365 amino acids; 7e-160) and Sm81P50 *versus* SM39 Serralysin (Accession WP_041035877) produced 60% identity (196 out of 329 amino acids; 5e-126).

## 3. Discussion

Five *S. marcescens* strains were isolated from the hemocoel of diseased *Phyllophaga* spp larvae. It was shown that the five isolates were able to cause an antifeeding effect in and mortality of *P. blanchardi* larvae after oral inoculation of bacteria. The antifeeding effect was not related to a taste aversion to the large dose of bacteria ingested, since control larvae fed with similar amounts of non-pathogenic bacteria did not show such an antifeeding effect. The antifeeding effect was only observed during the period from Day 7–11 after oral inoculation. It is possible that the toxic factors might take several days to damage the gut cells to produce the anti-feeding effect. It is also possible that during the inoculation period (Days 1–6) larvae might be attracted to some unknown attractants present in the cell-free culture supernatants. Third instar *Phyllophaga* larvae are extremely voracious at this stage, and the anti-feeding effect might be masked by the attractants during the inoculation period. Supporting this hypothesis is the fact that several enterobacterial volatile compounds working as semiochemicals able to attract insects have been reported [14]. During the 7–11 day period the carrot alone is not attractive enough and the anti-feeding effect is observed.

Bioassays were done with cell-free culture supernatant from two of the *S. marcescens* strains (Sm81and Sm65) hypothesizing that the bacteria might secrete toxin-like factors to the media. It was shown that the supernatant was able to cause an antifeeding effect and mortality either if the supernatant was ingested (Sm81) by or injected (Sm81 and Sm65) into the larvae. Then, a toxin-like factor seemed to be present in the culture supernatants of both isolates. As the toxic activity was depleted once the supernatant was boiled, it was suggested that the toxin-like factor might be of protein nature. In a next step, a purified 50 kDa Serralysin-like protein with protease activity, isolated from Sm81 cell-free culture supernatant, was shown to be able to cause mortality in *P. blanchardi* larvae. Serralysin-like proteins were identified in other *Serratia* strains by blast search. It is must be remarked that Serralysins reported to be insecticidal in the database showed 97%–99% identity to Sm81P50. Contrarily, Serralysin with no insecticidal reference presented lower homologies of 56%–63%. Further research is important to define the insecticidal potential of other Serralysin-like proteins, which it will be the topic of another publication.

It was observed that the five Serratia isolates produced an extracellular single 50 kDa protease. It is possible that the antifeeding effect and mortality induced by the five isolates was associated with the protease common to the five strains. A protease semi quantitative assay showed that Sm81 and Sm65 produced lower protease activity (which is an indirect form to measure the amount of protease), than Sm89, Sm67 and Sm73. However the apparent protease activity of each strain did not correlate with their ability to induce an antifeeding effect and mortality. Sm81 and Sm65 induced similar protease activities; however, their ability to induce mortality was different in oral bioassays with bacteria. Cell-free supernatants containing similar protein concentrations induced similar mortality in injection bioassays. It is possible that other virulence factors are involved in the pathogenic symptoms observed in bioassays done with bacteria. It is also possible that “similar” proteases of 50 kDa might contain different protein domains determining different toxicity degrees. It is possible that, for toxic activity, certain key amino acids might be essential. Further characterization of the individual Serralysin-like proteins will be necessary to define their role in toxicity.

The symptoms of infection and toxic activity are similar to the ones observed for *S. entomophila* Mor4.1 towards *Phyllophaga* spp larvae [4]; that is, an antifeeding effect very soon after oral inoculation and significant mortality at a later period of infection. Also similar to *S. entomophila* Mor4.1 is the extracellular production of toxin-like factors by the *S. marcescens* strains. The mortality observed caused by the *S. marcescens* isolates was observed after 27 days from oral inoculation of bacteria. However, a significant mortality was observed very soon (48–96 h) after intracoelomic inoculation of any of the five *S. marcescens* isolates, suggesting that the insect gut is a good barrier for orally infectious bacteria, and once the bacteria have invaded the hemocoel, there is the potential to surpass the insect immune system very soon after infection and kill the host. Apart from this, the sole presence of cell-free culture supernatants from the isolates Sm81 and Sm65 in the hemocoel caused a significant mortality of the larvae after five days from inoculation showing that the broths contain toxin-like factors able to kill the larvae.

No symptoms of infection were observed in the lepidoptera insect *Spodoptera frugiperda* by oral inoculation of bacteria suggesting that the pathogenic activity might be selective at least for *Phyllophaga* spp, that is, *P. blanchardi* and *P. ravida* since the *S. marcescens* strains were isolated from larvae belonging to the latter and evaluated in *P. blanchardi*. However, we do not know whether the Sm81 cell-free supernatant and the 50 kDa protein are toxic for *S. frugiperda* larvae. It is very important to establish the potential of the *S. marcescens* extracellular factors that kill different insect species since it would be desirable to use them alone or in combination with other bacterial toxic factors to potentiate their effects.

Since heat denatures proteins and the toxic activity of the Sm81 and Sm65 cell-free supernatant had significant decrements once the broths were boiled, it was suggested that the nature of the toxin-like factors in the *S. marcescens* cell-free broth was proteinaceous. The fact that the purified 50 kDa protein is insecticidal towards *P. blanchardi* supports this hypothesis. However, it is possible that other factors different to proteins might be implicated in the toxic activities of the bacteria, since it has been shown that bacterial wall components such as lipopolysaccharides might play an important role in pathogenicity to insects [15] and might be toxic to *P. blanchardi* larvae [16].

It was reported that the Serralysin-like proteins present protease activity [9]. *Serratia* proteases have been associated with bacterial virulence in vertebrates and have been proven to be insecticidal to grassland locust [9]. Although *S. marcescens* has very often been associated with insects, reports on the isolation and evaluation of pathogenicity of *S. marcescens* isolates on scarab larvae are scarce. Several strains of *Serratia* spp [17,18] and specifically, *S. marcescens* spp have been isolated from scarab larvae [19] but bioassays to evaluate pathogenicity have not been reported. The evaluation of the virulence of the *S. marcescens* 363 isolate by injection bioassay towards larvae of *Costelytra zealandica* showed that 1355 bacteria per larva caused a mortality of 96% after 6 days of injection [20]. In this report we show that a similar amount of bacteria (10^3^ cells) kill 100% of the larvae after 48 h to 96 h after inoculation, suggesting a stronger insecticidal activity of the Mexican *S. marcescens* isolates compared to the *S. marcescens* 363.

The possibility to use the strains of *S. marcescens* in a classical augmentative biological control program is discarded since it has been shown that this species of bacteria is able to cause infections in humans and animals [7] and therefore the liberation of the bacteria in high quantities in the field would be a potential risk to the environment. However, it has been suggested that the genus *Serratia* might be a source of genes and proteins for toxins and other virulence factors that could help to reduce crop damage or control insect pest [4]. This approach in the search of new toxins from entomopathogenic bacteria is becoming a strong directing force in the field of insect pest management worldwide [21]. The identification of the insecticidal activity associated with the 50 kDa protein is a good example. This is the first report on the identification of Serralysin-like protein with insecticidal activity towards *P. blanchardi* larvae.

## 4. Experimental Section

### 4.1. Culture Media and Strains

Nutrient broth or nutrient broth agar were used to grow bacteria at 30 °C. Bacteria were maintained in Luria-Bertani broth and 25% glycerol at −80 °C. The isolation of bacteria was done as reported [4,5]. Briefly, larvae of *Phyllophaga* spp showing disease symptoms were first washed with 15% sodium hypochlorite and sterile H_2_O. The bacterial isolation was done by puncturing the larval body to extract 20 µL from the body fluid, which was spread on nutrient broth agar and incubated over night. Single colonies were selected, propagated and used for bioassays and for further identification of bacterial species. Milk plates were used as indicators for protease activity prepared as reported [10]. Briefly, skim milk (10% wt/vol; commercial preparation) was dissolved in H_2_O and autoclaved for 10 min. The milk solution was cooled at 37 °C and 100 mL were added to autoclaved Luria-Bertani medium (500 mL), 2% agar, prior to overlay onto plates.

### 4.2. Biological Material

*P. blanchardi* larvae for bioassays were collected from cornfield soils within remnants of a pine forest at the community of Buena Vista del Monte (northern Morelos). All larvae were lodged individually in trays with soil from the original locality where the larvae came from and fed with small pieces of fresh carrot. Adult beetles emerging from field-collected larvae maintained under laboratory conditions were used for taxonomic identification as indicated [22]. Taxonomic identification of different larval instars was made as described [23]. Rearing *Spodoptera frugiperda* larvae for some bioassays (Lepidoptera: Noctuidae) was at Centro de Investigación en Biotecnología, Universidad Autónoma del Estado de Morelos (Cuernavaca, Morelos, Mexico) as reported [24], maintaining the larvae laboratory colony on a rice flour-based semi-synthetic diet.

### 4.3. Cell-Free Culture Supernatant

Bacterial cultures were centrifuged at 12,000× g for 10 min, and supernatants were filter sterilized through polyethersulfone 0.22 µm membranes (Millipore Corp., Bedford, MA, USA). The protein concentration was measured by the method of Bradford [25] using the commercial product Bio-Rad Protein Assay kit II. The cell-free culture supernatants were stored at 4 °C no longer than 2 weeks to be used for bioassays.

### 4.4. Electrophoretic Procedure

Proteins were analyzed by sodium dodecyl sulfate polyacrylamide gel electrophoresis (SDS-PAGE) prepared by conventional method as reported [26]. For zymography, a SDS-PAGE containing gelatin as substrate for protease was basically done as reported [11]. The resolving gel was prepared containing 0.1% gelatin. After SDS-PAGE, the gel was washed 1 h in TritonX-100 at room temperature to remove SDS and restore enzyme activity. Then it was incubated overnight at 37 °C in 0.1 M Tris-HCl pH8. The gel was stained in Coomassie blue solution as reported [26]. In some cases proteins were concentrated by ultra-filtration using 3-kDa-molecular-mass-cutoff membrane (Millipore Corp., Bedford, MA, USA) and adjusted to the desired concentration with H_2_O.

### 4.5. Evaluation of Protease Activity on Milk Plates

Single bacterial colonies were grown for 24 h on agar-plates containing milk. The diameter of milk clearing zones resulting from hydrolysis of milk casein was measured. The difference of the diameter size of five colonies per strain was evaluated by ANOVA. For cell-free culture supernatants, bacterial cultures were grown for 24 h at 30 °C shaking at 250 rpm and adjusted to an OD_540_ of 2.3. Five µL of cell-free culture supernatant were placed on milk-agar plates and incubated at 37 °C for 24 h. The diameter of halo size was measured and the difference of the diameter size of five samples per strain was evaluated also by ANOVA.

### 4.6. Bioassays with Insect Larvae

Oral inoculation tests were done basically as reported [4] by feeding the larvae during six consecutive days (inoculation period) as indicated in the figure legend with small cylinders of carrot (approximately 16 mm^3^ each) containing a dose of 10^8^ bacteria per larva or cell-free culture supernatant obtained from the bacterial strains grown for 24 h (2 µg of protein per larvae). The carrot cylinders were previously dehydrated in an oven at 60 °C during 2 h. Then, the pieces of carrot were individually located in plastic containers and re-hydrated with 50 µL of bacterial culture or cell-free culture supernatant or LB plus BSA, containing the bacteria or the proteins at the desired concentration. Control larvae were fed with uncoated pieces of carrot and/or carrot coated with the non-pathogenic bacteria *Serratia plymuthica* ATCC15928, or carrot coated with nutrient broth containing BSA. The piece of carrot was changed every day. After the inoculation period, the larvae were fed with uncoated carrot. Tests were also done with carrot coated with cell-free broths from *E. coli*Top10 culture. Differences in the numbers of larvae showing disease symptoms were estimated by hypothesis test with two-way frequency tables, using the χ^2^ statistical test or Fisher exact test. The mortality and the amount of food consumed were recorded for each larva at 24 h intervals. To analyze the results, the larvae from different treatments were arranged in groups according to proximity in the percentage of mortality to detect statistical differences between groups for the χ^2^ test. The maximum percentage of mortality observed at the shortest time in days when the differences between the evaluated groups of larvae are statistically significant is reported. Unless otherwise stated in the figure legend, groups of larvae analyzed by the χ^2^ test are shown by different letters above the bars or lines in the figures. To evaluate differences in the proportion of larval feeding, the percentage of food consumed was arcsine transformed and analyzed by ANOVA and multiple comparisons were done using the Tukey-Kramer (*p* < 0.001) test using the software Graph Pad 3.10 (Graph Pad Software, Inc., La Jolla, CA, USA). For all the bioassays, data are representative of at least two independent bioassays. For oral bioassays with *S. frugiperda* larvae, 35 µL of broth containing 10^8^ bacteria mL^−1^ were added to the surface of the artificial diet previously poured into plastic containers (2 cm^2^), where neonate (L1) *S. frugiperda* larvae were individualized (*n* = 24).

Bioassays by injection with *P. blanchardi* were done by administering 50 µL of bacterial suspension containing 10^3^ bacteria per larva or 50 µL of cell-free culture supernatant (2 µg of protein per larva), directly into the hemocoel, while control larvae were injected with 50 µL of nutrient broth containing BSA (2 or 50 µg of protein per larva) or cell free culture broth from an *E. coli* Top10 (Invitrogen™) and/or *E. coli* Epi300 (Epicenter) culture with similar protein concentration. Mortality was recorded at 24 h intervals. The insects used for oral or injection bioassays were healthy third-instar *P. blanchardi* larvae. All bioassays were done using 10 to 14 larvae per treatment. Neonate larvae of *Spodoptera frugiperda* (Lepidoptera: Noctuidae) were used for an oral bioassay done to test the five isolates of *S. marcescens* as described by Aranda *et al.* [27]. For this purpose, a bacterial suspension (10^3^ bacteria per larva) of the different isolates was applied to the diet surface.

For experiments to evaluate dose-dependency of Sm81P50, the activity of the purified Sm81P50 was tested against *P. blanchardi* larvae, using a dose range of 1–8 µg protein per larva. Data were fitted to the probit curve using the software PoloPlus 1.0 (LeOra software, 1987).

### 4.7. Bacterial Identification

For molecular identification, amplification of the 16S rRNA gene of the different isolates was done using Taq DNA polymerase (Fermentas) from the bacterial genomic DNA. The amplification product of 1.5-kb DNA was purified from an agarose gel (1%), using the commercial product for DNA purification, PureLink quick extraction kit (Invitrogen). The PCR product was sequenced at the automated sequencing facilities of the Instituto de Biotecnología, UNAM (Cuernavaca, Mexico). Universal primers directed toward the 16S rRNA gene were as reported [4]. The forward primer used was 5’ CGG AATTCA GAA GTT TGA TCM TGGMCTC AG 3’, and the reverse primer was 5’CGG GAT CCA AGG AGG TGA TCC ANC CRC A 3’. For phenotypic and biochemical tests to taxonomically identify the bacterial isolates, we used the characteristics described for *Serratia* spp by Grimont and Grimont [6].

### 4.8. Purification of the 50 Kda Serralysin-Like Protein

The bacterium *S. marcescens* 81 was grown in nutrient-broth for 24 h at 30 °C with rotary shaking at 250 rpm. The bacterial culture was centrifuged (12,000× g, 30 min, 4 °C) and the supernatant was filter sterilized through a 0.22 polyethersulfone membrane. Proteins present in the supernatant were precipitated with ammonium sulfate at 80% saturation. The precipitate was resuspended in 20 mM Tris-HCL (ph8.0), 200 mM NaCl. After dialysis, the proteins were loaded onto a Q-Sepharose anion-exchange 1 ml column (HiTrap^TM^ Q Fast Flow, GE Healthcare Bio-Sciences). The same buffer was used to equilibrate and wash the column to remove unbound material. A first step of 200 mM·NaCl was used to remove contaminant proteins. Serralysin-like protein was eluted with 300 mM·NaCl. The purified protein was dialyzed against phosphate-buffered saline (PBS). To determine purity, a sodium dodecyl sulfate-polyacrylamide gel electrophoresis was performed. The protein concentration was determined by the method of Bradford [25] using the commercial product Bio-Rad Protein Assay kit II.

The identity and amino acid sequence of the Serralysin-like protein were determined using tandem-mass spectrometry (LC-MS/MS) at the automated facilities of the Instituto de Biotecnología, UNAM (Cuernavaca, Mexico). The samples were first reduced with ditiotreitol (DTT) and alkylated with iodoacetamide (Sigma-Aldrich). Then they were digested *in gel* with trypsin (Promega Sequencing Grade Modified Trypsin). The resulting peptides were applied in a Liquid chromatography-mass spectrometry (LC-MS) with a nanoflux bomb EASY-nLCII (Thermo-Ficher Co. San Jose, CA, USA), a mass spectrometer LTQ-Orbitrap Velos (Thermo-Ficher Co., San Jose, CA, USA) and a nano-electrospray (ESI) ionization system. The spectrometer was calibrated with a Calmix solution (N-butilamina, cafein, Met-Arg-Phe-Ala) and Ultramark 1621. For LC, a 10%–80% gradient of solution B (water/acetonitrile 0.1% formic acid) was used during 120 min using a home made capillar column (0.75 µm diameter × 10 cm large RP-C18) with a flux of 300 nanoliters per minute. Collision-Induced Dissociation and High Energy Collision Dissociation methods were used for the peptide fragmentation, selecting charged ions of 2^+^, 3^+^ and 4+. Ions with charge 1+ and above 5+ as well as undefined charges were not considered. Positive detection mode was used in all specters. Capture and performance of fragmentation data were done according to the total ion scanning and predetermined charge with 3.0 (*m*/*z*) isolation width, a collision energy of 35 arbitrary units, an activation Q of 0.250, an activation time of 10 millisecond and a maximum injection time of 10 millisecond per micro-scanning. The automatic capture of data was done using ion dynamic exclusion: (i) exclusion list of 400 ions; (ii) pre-exclusion time of 30 s and (iii) exclusion time of 300 s.

Mass spectrometry data were used for a search in the database of the National Center for Biotechnology Information (NCBI) [8] with the program ProteinProspector (University of California, San Francisco, CA, USA). Also, a restricted search for *Serratia* in the Protein Data Bank of Europe, UniProt using Proteome Discoverer1.4 software was done.

## 5. Conclusions

In conclusion, this study shows the isolation and characterization of five isolates of *S. marcescens* that are pathogenic for *P. blanchardi* larvae. The *S. marcescens* isolates cause different degrees of antifeeding effect and mortality and the Sm81 cell-free broth is also able to cause similar effects as the bacteria, either by oral or injection bioassays. In addition, it was shown that both pathogenicity signs were abolished when the broth was boiled, presumably because of protein denaturation and consequent loss of toxic activity suggesting a role of *Serratia* extracellular proteins in toxicity against insects. A Serralysin-like 50 kDa protein was found to be insecticidal toward *P. blanchardi* larvae supporting the hypothesis. It is proposed that the *S. marcescens* isolates might be useful as a source for genes and toxins with biotechnological applications to prevent crop damage caused by *Phyllophaga* spp. Apart from this, it is very important to characterize the 50 kDa insecticidal protein to determine its potential as an insect biocontrol agent.

## Supplementary Files

Supplementary File 1
